# The Surgery of the Chest

**Published:** 1896-01-11

**Authors:** 


### PROGRESS IN SURGERY.

### THE SURGERY OF THE CHEST.

The surgery of the lungs has recently been reviewed by Reclus at the French Congress of Surgery.[1] He thinks that surgical intervention in traumatic hæmorrhage from the lung is rarely called for, but that when it is, the thoracic wall should be freely opened so as to lay bare the wound and permit of ligature of the bleeding vessel or tissue, or packing with iodoform gauze. Dr. Quénu recorded a case of penetrating wound of the chest in which hæmorrhage from the injured lung necessitated a free opening of the pleural cavity and exposure of the wound in the lung. But the operation was not undertaken until more than a week after the injury, and was, apparently, required, not so much to stop the bleeding as to empty the pleural cavity of clot. The wound in the lung was, however, packed with iodoform gauze, and the patient recovered. Dr. Michaux also reports a case of bullet wound of the lung, in which the wound in the chest wall was at once closed, but signs of considerable hæmorrhage into the pleura set in, and the patient became somewhat collapsed. A free opening of the pleural cavity revealed a litre of blood there, and exposed the wound in the lung, which was plugged with success. Dr. Michaux considers that surgical intervention is needed in such cases, as Nélaton has shown that the mortality in cases of extensive extravasation of blood of pleuro-pulmonary origin is at least 50 per cent.

Attempts have been made to aspirate pulmonary apoplexy and œdema, but without success. Excision of portions of tuberculous lung does not seem to have been performed lately. The tendency to dissemination in this disease seems to contra-indicate the attempt to remove any one focus, and the results in most of the few published cases have been disastrous; but at the congress Dr. Tuffier presented the patient from whom he had excised the localised tuberculous deposit in the apex of the lung four and a half years before. He remained quite well, and no indication of pulmonary tubercle was present. The diseased lung tissue had been excised through the second interspace and no rib resected. By detaching the parietal pleura a kind of extra pleural pneumo-thorax had been formed, and the apex of lung was drawn out through the wound and thoroughly examined. The tuberculous lesion was the size of a walnut and was excised together with two centimetres of healthy tissue below it. It was found, on careful microscopic examination, to be tuberculous. Respiration at the apex operated on—at the time Dr. Tuffier showed him—did not differ from that at the other apex. Although agreeing with Dr. Reclus that only very few cases indeed of pulmonary tubercle were fit for operation, he yet thought that now and then a case like his own might be operated on with advantage.

The indications for drainage of tuberculous or bronchiectatic cavities Reclus considers are extremely rare. He agrees with Truc that these indications are (1) that the pulmonary excavation constitutes the principal lesion; (2) that symptoms of septic absorption predominate, determining pyrexia; (3) when the patient coughs a great deal and is exhausted by copious expectoration. For hydatic cyst of the lung incision and drainage has, he thinks, been proved to be the most successful methods of treatment. For pneumotomy to be successful in cases of gangrene of the lung, the gangrene must of course not be diffuse. When the lesion though circumscribed is of large extent, and when the putrefactive matter does not flow out freely, but causes symptoms of septic absorption, the exact seat of the mischief should be carefully determined, and the cavity opened as soon as it has formed. In the treatment of abscess of the lung he considers that incision and drainage has been as successful as in the treatment of hydatid cysts. His statistics of reported cases show a recovery of 20 out of 23 cases. Most of the abscesses followed pneumonia, a few typhoid fever, and three were suppurating hydatic cysts. Out of four cases which were pyæmic in origin three recovered. In draining a pulmonary cavity he thinks portions of the ribs should be removed over a fairly large area—in fact an Estlander's operation should be performed to allow the sides of the cavity to fall together. If the pleura is not adherent over it, the layers must be stitched together, and the incision into the cavity should be made with a cautery rather than a knife, as fatal hæmorrhage has followed the use of the latter instrument. As soon as the aperture has been made, the finger is introduced into the opening in order to enlarge it. Reclus is opposed to the practice of flushing the cavity, as in one case the injection of a solution of boracic acid and thymol determined inflammation of the laryngo-bronchial tract and speedy death. Péan thinks[2] solid tumour of the lung should be extirpated when superficial, but considers that the occasions for this treatment must be rare, as such growths are almost always secondary.

**The Treatment of Serous Pleural Effusion by Incision and Drainage.**—Mr. Rutherford Morrison records[3] a case of serous pleural effusion in which the fluid re-formed again and again after aspiration, but was successfully treated by incision and drainage with every aseptic precaution. The case occurred before Dr. West's (published earlier in the year[4]), in which free incision and drainage proved successful after the failure of repeated aspiration. Mr. Morrison adopted the method because of the success which he had seen in drainage of serous effusion into the knee joint in Sir Joseph Lister's hands. Dr. Albert Wilson[5] also reports some cases of serous pleural effusion treated in the same way, but paracentesis was not employed, and would probably have proved as successful. Dr. West strongly condemns incision and drainage in such cases, reserving it for those only in which aspiration fails, as he considers that by incision a simple serous effusion may be converted into an empyæma, and that the treatment by paracentesis is usually very successful, the lung expanding well unless operation is too long delayed.

**The Mode of Healing of Empyæma** is discussed by Mr. Bilton Pollard.[6] He rejects the theory of Roser that adhesion of the lung to the parietal pleura is brought about by granulation commencing at the angles where the two layers of pleura are in contact, and thence extending over the whole surface of the lung; but considers Weissgerber's theory probably correct. According to this theory, the contact of the two surfaces which must take place before adhesion can occur, is due to expansion of the collapsed lung from coughing, which raises the pressure of the air inside the lung above that on the outside, the entry of blood into the vessels of the alveoli assisting in maintaining the increase of bulk. The dressing over the wound in the chest wall acts as a fairly perfect valve, making the entrance of air in inspiration difficult. When the lung, expanded by coughing, comes in contact with the chest wall, the two layers are held together by a kind of sucker-like action until adhesions have formed. This sucker-like action was proved to exist in some cases of surgical emphysema due to injury of the lung by broken rib, by the absence of pneumothorax. Mr. Pollard does not advise washing out the pleural cavity, even in cases of fetid empyæma, as he thinks the discharge quickly becomes sweet with simple drainage. If the lung does not expand well after an empyæma is incised and drained, he recommends the inhalation of compressed air, or raising the intra-pulmonary pressure by making the patient cough, or causing him to play upon wind instruments. In double empyæma Mr. Pollard advocates simultaneous drainage of both pleuræ.

**Subphrenic Abscess.**—The etiology and diagnosis of this disease is discussed by Lampe,[7] who considers that the origin of the largest number of cases is from the stomach, in consequence of perforation of a gastric ulcer or in rare cases of carcinomatous ulceration. If due to perforation of a gastric ulcer the abscess will usually be situated in the left subphrenic space, but those following perforation of a carcinomatous ulcer will be on the right side because the pylorus is most frequently the seat of this form of neoplasm. Of thirty-five cases of subphrenic abscess due to perforation of the stomach twenty-five who were not operated on all died, whilst of ten operated on only three recovered. In subphrenic abscess from perforation of the vermiform appendix the pus either creeps along behind the meso-cæcum in the cellular tissue which is continuous with the perinephritic and subphrenic cellular tissue, or the veins of the appendix and cæcum becoming infected start pyæmic abscess in the liver with secondary subphrenic abscess. A case in which an abscess spread from the cæcal region into the subphrenic, due to gangrene of the appendix, is related. In thiee of subphrenic abscess there was serous effusion into the pleural cavity above the abscess. If by exploratory puncture in an intercostal space clear fluid is withdrawn, and if pus is found at a puncture lower down, it indicates the almost certain existence of subphrenic abscess, though it is just possible there might be a serous effusion in the pleura around a localised empyæma. Lampe, therefore, considers that there is no absolutely sure sign of subphrenic abscess. In the cases he describes, gas formation had not taken place in the abscess cavity, so that this valuable diagnostic sign was absent.

[1] Medical Week, Oct. 25, 1895, p. 505; Nov. 15, p. 548; and Nov. 1, p. 521. [2] Presse Méd., Oct. 23, and B. Med. J. Epitome, Dec. 7. [3] Brit. Med. Journal, July 13, p. 75. [4] Ibid, April 27. [5] Ibid, Nov. 2. [6] Ibid, Nov. 2, p. 1,131. [7] Am. J. of Med. Sciences, Sept., 1895, p. 347, and Lampe Müncheuer Medicinische Wochenschireft, 1895, No. 20.

